# Morphine Exacerbates HIV-1 Tat-Induced Cytokine Production in Astrocytes through Convergent Effects on [Ca^2+^]_i_, NF-κB Trafficking and Transcription

**DOI:** 10.1371/journal.pone.0004093

**Published:** 2008-12-31

**Authors:** Nazira El-Hage, Annadora J. Bruce-Keller, Tatiana Yakovleva, Igor Bazov, Georgy Bakalkin, Pamela E. Knapp, Kurt F. Hauser

**Affiliations:** 1 Department of Pharmacology and Toxicology, Virginia Commonwealth University School of Medicine, Richmond, Virginia, United States of America; 2 Division of Basic Research, Pennington Biomedical Research Center/Louisiana State University, Baton Rouge, Louisiana, United States of America; 3 The Division of Biological Research on Drug Dependence, Department of Pharmaceutical Biosciences, Uppsala University, Uppsala, Sweden; 4 Department of Anatomy and Neurobiology, Virginia Commonwealth University School of Medicine, Richmond, Virginia, United States of America; 5 Institute for Drug and Alcohol Studies, Virginia Commonwealth University, Richmond, Virginia, United States of America; New York University School of Medicine, United States of America

## Abstract

Astroglia are key cellular sites where opiate drug signals converge with the proinflammatory effects of HIV-1 Tat signals to exacerbate HIV encephalitis. Despite this understanding, the molecular sites of convergence driving opiate-accelerated neuropathogenesis have not been deciphered. We therefore explored potential points of interaction between the signaling pathways initiated by HIV-1 Tat and opioids in striatal astrocytes. Profiling studies screening 152 transcription factors indicated that the nuclear factor-kappa B (NF-κB) subunit, c-Rel, was a likely candidate for Tat or Tat plus opiate-induced increases in cytokine and chemokine production by astrocytes. Pretreatment with the NF-κB inhibitor parthenolide provided evidence that Tat±morphine-induced release of MCP-1, IL-6 and TNF-α by astrocytes is NF-κB dependent. The nuclear export inhibitor, leptomycin B, blocked the nucleocytoplasmic shuttling of NF-κB; causing p65 (RelA) accumulation in the nucleus, and significantly attenuated cytokine production in Tat±morphine exposed astrocytes. Similarly, chelating intracellular calcium ([Ca^2+^]_i_) blocked Tat±morphine-evoked MCP-1 and IL-6 release, while artificially increasing the concentration of extracellular Ca^2+^ reversed this effect. Taken together, these results demonstrate that: 1) exposure to Tat±morphine is sufficient to activate NF-κB and cytokine production, 2) the release of MCP-1 and IL-6 by Tat±morphine are highly Ca^2+^-dependent, while TNF-α appears to be less affected by the changes in [Ca^2+^]_i_, and 3) in the presence of Tat, exposure to opiates augments Tat-induced NF-κB activation and cytokine release through a Ca^2+^-dependent pathway.

## Introduction

Among human immunodeficiency virus type 1 (HIV-1)-infected individuals, injection drug users are at greater risk than nonusers of developing HIV-associated neurological impairment, as well as other opportunistic infections [1]–[3]. Not only does injection drug use provide a mode for viral spread, but the opioid system (i.e., endogenous opioid peptides and receptors) also plays a fundamental role in modifying, and in many cases exacerbating the pathogenesis of neuro-acquired immune deficiency syndrome (neuroAIDS) [4]–[6]. Brain regions expressing a high number/density of μ-opioid receptors (MOP), such as the striatum and the hippocampus, have increased viral loads and are preferentially decimated by HIV infection (reviewed in [4]–[6]). Therefore, the central nervous system (CNS) may be uniquely susceptible to the combined effects of opiate substance abuse and HIV-1. μ-Opioid receptor-expressing astrocytes in particular may be an important site of convergence for opiate drug-HIV-1 actions. Previously, we and others have shown that opiates exacerbate the astroglial response to HIV-1 proteins [7]–[9], causing increased disruptions in [Ca^2+^]_i_ homeostasis and increased chemokine release. However, the mechanisms underlying opiate-induced increases of chemokine expression and release in astrocytes exposed to HIV-1 protein are incompletely understood.

Astrocytes are essential in regulating neuronal function and support, and participate in neurogenesis [10], synaptic transmission [11], brain repair [12], and in the formation and preservation of the blood-brain barrier [13]. When brain homeostasis is disrupted, astrocytes become activated, producing a variety of factors, including nitric oxide, neuropeptides and cytokines [14], [15]. Astrocytes are a major source of cytokines (e.g., TNF-α and IL-1β) and chemokines (e.g., CCL2/MCP-1, CCL5/RANTES, and CCL3/MIP-1 α) in the HIV-1 infected CNS [14], [16]. Opiates act by exacerbating the astroglial response to HIV-1 [17], which involves the release of cytokines and chemokines, the recruitment of macrophages and activated microglia, and an amplification of the inflammatory response [18].

MCP-1, IL-6 and TNF-α are involved in the induction and perpetuation of innate immune and inflammatory responses. MCP-1 is a potent chemokine and is thought to be involved in the progression of neuroAIDS and HIV associated dementia by virtue of its ability to recruit and activate macrophages/microglia [19], [20]. MCP-1 appears to play a central role in promulgating neuroimmune disease processes within the CNS, as recent evidence indicates that MCP-1 levels are highly correlated with neurocognitive defects accompanying HIV-1 [21], [22]. Similarly, IL-6 and TNF-α are elevated in the central nervous system of individuals with AIDS or AIDS dementia complex [23]. IL-6 can upregulate production of HIV in infected monocytic cells and IL-6 can act synergistically with TNF-α [24]. Importantly, depending on concentration, morphine can significantly increase or decrease lipopolysaccharide-induced NF-κB transcriptional activity in murine macrophages, which is directly proportional to TNF-α and IL-6 release [25]. In light of evidence that the pattern of cytokine release in response to Tat and/or morphine differs in macrophages and astroglia [17], and since NF-κB along with other transcription factors induce the production of MCP-1, IL-6 and TNF-α by astrocytes [26]–[28], we were prompted to explore whether NF-kB was differentially regulated by Tat±morphine in astrocytes and determine whether opiates are acting via NF-κB to modulate cytokine production by HIV-1 Tat exposed astrocytes.

NF-κB is an essential transcription factor that plays a central role in the regulation of genes involved in the immune and inflammatory responses [29]–[31]. NF-κB is also involved in development, cell proliferation, apoptosis, signaling injury, synaptic transmission, neuronal plasticity, and neurodegenerative diseases [32], [33]. The NF-κB family is comprised of five members, RelA (p65), RelB, c-Rel, p105/p50, and p100/p52. All share the Rel homology domain, which allows their dimerization and translocation to the nucleus. Association with IκB, an inhibitor of NF-κB, tightly regulates the activity of NF-κB. NF-κB is inactive and retained in the cytoplasm when bound to the IκB proteins. However, NF-κB activation triggers events that generally result in (1) the polyubiquitination and degradation of IκB, (2) the unmasking of nuclear localizing signals (NLS), (3) the translocation of unbound NF-κB molecules to the nucleus where they modulate the transcription of specific genes [34], [35]. In the present study, we examined mechanisms by which opioids influence NF-κB signaling pathways and whether this regulates inflammatory gene activity in astrocytes exposed to HIV-1 proteins. Data presented here show how opioids modulate the regulatory mechanisms involved in the secretion of cytokines IL-6 and TNF-α, and in particular the production of the chemokine, MCP-1.

## Materials and Methods

### Materials

RPMI 1640 was purchased from Gibco (Grand Island, NY). Morphine sulfate was obtained from the National Institute on Drug Abuse (NIDA, Drug Supply System, Bethesda, MD). Tat_1–72_ recombinant protein was produced at the University of Kentucky Tat core facility. The NF-κB inhibitor, parthenolide, and the IκB inhibitor, Ro106-9920, were purchased from Sigma (St. Louis, MO), while leptomycin B (LMB) was purchased from LC Laboratories (Woburn, MA). Antibodies directed against β-actin, and p65 (RelA) were purchased from Santa Cruz Biotechnology (Santa Cruz, CA), and used for immunoblotting or immunocytochemistry.

### Cell Culture

Astroglial-enriched cultures were prepared using 1–4-day-old ICR mice (Charles River Laboratories, Charles River, MA) as previously described [36]. Briefly, mice were anesthetized and euthanized according to NIH and IACUC guidelines, which minimizes the number of animals used and their discomfort. Striata were aseptically isolated, minced in media, incubated with trypsin/DNase (37°C, 30 min), triturated through a series of decreasing bore pipettes, and filtered sequentially through 130 µm pore nitex filters. Cells for each experiment were pooled from two to four striata. Growth medium favoring astroglial enrichment consisted of Dulbecco's Modified Eagle's Medium (DMEM) (GIBCO) supplemented with glucose (27 mM), Na_2_HCO_3_ (6 mM), HEPES (10 mM), and 10% (v/v) fetal bovine serum (FBS; JRH Biosciences or Hyclone, Logan, UT). Cells were plated at 50,000–100,000 cells/cm^2^ in poly-L-lysine-coated 12 or 24-well plates for protein detection (Costar, Corning Life Sciences, Acton, MA), or in 100 mm culture dishes for transcription factor assays. Cells were grown for 7–10 days until they reached 70–80% confluency at 37°C in 5% CO_2_/95% air at high humidity. Cultures consisted of >98% astrocytes as determined by staining for glial fibrillary acidic protein (GFAP). Unless mentioned elsewhere, cell culture products were purchased from Invitrogen (Gaithersburg, MD).

### Experimental Treatments

Recombinant Tat_1–72_ was produced and purified as described previously [17]. Briefly, the *tat* gene encoding the first 72 amino acids of HIV-1_BRU_ (obtained from Dr. Richard Gaynor, through the NIH AIDS repository) was inserted into an *Escherichia coli* Pin Point Xa-2 vector (Promega, Madison, WI). Biotinylated Tat was purified on a column of soft release avidin resin, cleaved from the fusion protein using factor Xa, eluted and desalted using a PD10 column, and treated with Pierce Detoxi-Gel (Pierce). A reticulocyte amoebocyte lysate assay is used to assay for trace endotoxin (Associates of Cape Cod, Inc.; sensitivity<1 pg/ml). If endotoxin is detected, the Tat is discarded. Cytokine release was not observed in astrocytes treated with non-toxic, deletion mutant Tat (Tat_Δ31–61_) or immunoneutralized Tat [9], [17].

Cells were continuously exposed to medium alone (vehicle-treated controls), morphine sulfate and/or Tat_1–72_ for varied intervals. The concentrations of morphine and Tat_1–72_ were 500 nM and 100 nM, respectively. Multiple concentrations of inhibitors were initially screened as part of the intracellular calcium studies [17], [37]. For each inhibitor, the initial range of concentrations screened was based on published values. Parthenolide (0, 1, 5, 10 and 20 µM) was used to inhibit NF-κB activation. BAPTA/AM (0, 1, 5, 10 and 20 µM) was used to chelate calcium. Leptomycin B (0, 1, 5, 10 and 20 nM) has been shown by others to prevent export of proteins from nucleus to cytoplasm, while Ro106-9920 (0, 1, 5, 10 and 20 µM) has been shown to inhibit proteasome activity and IκBα degradation. For some experiments, inhibitors were added to cell cultures and preincubated for 15–20 min at 37°C (5% CO_2_/95% air), followed by 6, 12 and 24 h of continuous incubation with Tat±morphine as previously described [17].

### Transcription Factor Arrays

We used TranSignal™ Protein/DNA Arrays I & II (Panomics Inc., Redwood City, CA) to screen for potential changes in activation of a wide spectrum of transcription factors due to Tat±morphine exposure. Astrocytes were harvested after 4 or 24 h treatment. Nuclear extracts of control and treated astrocytes were prepared using a Nuclear Extraction Kit (Panomics) and were incubated with a biotin-labeled, DNA-binding oligonucleotide probe mixture (15°C for 30 min) provided with the array kit to allow formation of transcription factor-DNA complexes. Complexes were isolated on a 2% agarose gel. Probes were then separated from transcription factors and hybridized to TranSignal Array membranes, which contain consensus-binding sequences for the DNA probes in the mixture. HRP chemiluminescence detection was used to visualize biotinylated sequences bound to each array. A Kodak Image Station 440CF with 1-D software was used for densitometric quantitation of transcription factor levels.

### Real-Time PCR

For real-time RT-PCR, total RNA was isolated from treated cells using GenElute™ Mammalian Total RNA kit (Sigma). cDNA was synthesized from 2 µg of total RNA using a High-Capacity cDNA Archive Kit (Applied Biosystems, Warrington, UK). Standards containing varying concentrations of cDNA templates (cDNA mix and 2×, 4×, 8×, 16× dilutions) were used to generate a standard curve. TaqMan Universal PCR Master Mixes and gene-specific TaqMan probes and primer sets for MCP-1 and 18S rRNA was purchased from Applied Biosystems. Real time RT-PCR was conducted using the Prism 7500 system (Applied Biosystems). MCP-1 mRNA was normalized to 18S mRNA. Primers for IL-6 and TNF-α were purchased from Integrated DNA technologies (IDT) and normalized to β-actin mRNA.

### Electrophoretic Mobility Shift Assay (EMSA)

All procedures for nuclear protein extraction were performed at 4°C with ice-cold reagents. Cells were gently scraped in PBS, washed, and resuspended in 500 µl of lysis buffer A [15 mM KCl, 10 mM HEPES, 2 mM MgCl_2_, 0.1 mM EDTA, 1 mM PMSF, 1 mM dithiothreitol (DTT), 10 µg/ml aprotinin, 2 µg/ml leupeptin, 0.1% NP-40, pH 7.6]. Cell suspensions were then incubated for 10–15 min on ice with occasional vortexing, and centrifuged for 30 s to pellet the nuclei. Pelleted nuclei were rinsed with wash buffer B (2 mM KCl, 25 mM HEPES, 0.1 mM EDTA, 1 mM PMSF, 1 mM DTT, 10 µg/ml aprotinin, 2 µg/ml leupeptin, pH 7.6) and incubated at 4°C for 20 min. Nuclear extracts were then prepared by centrifugation at 20,000× *g* for 15 min in buffer C (25 mM HEPES, 0.1 mM EDTA, 20% glycerol, pH 7.6) and stored at −80°C until used for EMSA. The probe containing NF-κB-binding element was labeled with γ-[^32^P] (Amersham Life Science) (3000 Ci/mmol, 250 µCi) using T4 polynucleotide kinase (Boehringer Mannheim). 10 µg of extracts was incubated for 20 min with 20,000 cpm of [^32^P]-labeled double-stranded oligonucleotide at 4°C in a 25 µl reaction mixture containing: 10 µg/ml BSA, 10× buffer D (100 mM KCl, 20 mM HEPES, 0.5 mM EDTA, 2 mM DTT, 0.1 mM PMSF, 20% glycerol, 0.25% NP-40, pH 7.6), 5× buffer F (300 mM KCl, 100 mM HEPES, 10 mM DTT, 100 µM PMSF, 20% Ficoll, pH 7.6) and 1 µg/µl poly(dI-dC). In competition assays, 100× cold NF-κB competitors were added. The DNA–protein complex was separated on a non-denaturating 4% polyacrylamide gel in TBE buffer (Tris-HCl, boric acid, EDTA 2 mM, pH 8.0). After electrophoresis, the gel was dried and autoradiography performed by overnight exposure to X-ray film.

### Western Immunoblotting

Cells were grown in 24-well plates and all procedures for protein extraction were performed at 4°C with ice-cold reagents. Cells were washed in cold PBS, scraped, harvested and disrupted in RIPA buffer [1× PBS, 1% NP-40, 0.5% sodium deoxycholate, 0.1% SDS, plus protease inhibitor cocktail (Sigma)]. After 30–60 min incubation on ice, cell lysate was centrifuged for 20 min (15,000× g, 4°C). The supernatant fluid was collected and the protein concentration was determined by BCA protein assay kit (Pierce). Each lane was loaded with 20 µg samples. Blots were probed with the indicated antibodies and immune complexes were detected by enhancement chemiluminescence (Amersham Pharmacia Biotech). A Kodak Image Station 440CF with 1-D software was used for densitometric quantitation.

### Bio-Plex Kinase Assay

Antibodies directed against phosphorylated and non-phosphorylated forms of IκB, which were covalently coupled to internally dyed beads, were purchased from Bio-Rad (Hercules, CA) and added to a 96 well plate. Protein lysates from treated astrocytes were added to each well and the target kinases reacted with the coupled beads. After a series of washes to remove unbound protein, biotinylated antibodies specific for the phosphorylated and the non-phosphorylated forms of IκB were added to the reaction. Next, streptavidin-phycoerythrin (streptavidin-PE) was added to visualize the biotinylated antibodies on the bead surface. Data from the reaction were acquired using the Bio-Plex suspension array system, a dual-laser, flow cytometry-based microplate reader system (Bio-Rad, Hercules, CA). Data was presented as the ratio of p-IκB∶IκB, fluorescence signals obtained using Bio-Plex Manager™ software (Bio-Rad).

### Immunocytochemistry

After treatment, cells were fixed in 4% paraformaldehyde, permeabilized with 0.1% Triton-X, 0.1% BSA in PBS, and blocked with 1.5% goat serum in PBS (15 min. each procedure). Cells were incubated with anti-p65(RelA) monoclonal antibodies for 1 h at room temperature, followed by incubation with goat anti mouse Cy3 (Jackson Immunology). Nuclei were stained with 1 µg/ml Hoechst 33258 in PBS for 20 min. Fluorescent cells were visualized using Zeiss AxioVision Z1 microscope and Zeiss MRm digital camera.

### ELISA

ELISA plates (96-well) were coated with corresponding mouse antibodies (1–2 µg/ml) overnight at 4°C. Subsequent ELISA steps were conducted at room temperature. Plates containing the MCP-1 antibody were blocked with 1% bovine serum albumin (BSA) in PBS, while plates containing the IL-6 and TNF-α antibody were blocked with 1% BSA plus 5% sucrose in PBS for 1 h. Following 3 washes, each plate was incubated with culture supernatants and a series of dilution standards for 2 h. Capture antibody was added to each plate and incubated for 90 min. Horseradish peroxidase (1∶4000) was added to TNF-α and IL-6 coated plates, and each plate was incubated for 20 min. Tetramethylbenzidine substrate (BD Pharmingen) was then added for color development and microplates were read at 450 nm using Victor 3 plate reader (PerkinElmer Inc., Waltham, MA, USA) immediately after terminating the reaction.

### Statistical Analyses

Data were analyzed using one or two-way ANOVA. When significant main effects were identified (*P*<0.05), Duncan's multiple comparisons test was used to compare intergroup differences. If group variances were determined to be non-homogeneous, Kruskal-Wallis nonparametric ANOVA and multiple comparisons test were used to determine whether differences were statistically significant. Results given are at 6 h after Tat±morphine exposure unless noted otherwise.

## Results

### Tat and/or Morphine Modulates the Activity of Inflammatory and NF-κB/Rel Family Transcription Factors

Exposure to Tat±morphine caused alterations in 90 of the 152 transcription factors collectively surveyed on TranSignal™ Protein/DNA Arrays I and II. Many of the factors that were elevated were related to inflammation. Of these, we decided to pursue c-Rel because it was the only factor that showed approximate 2-fold increases in DNA binding activity at both 4 (1.91±0.13) and 24 h (1.85±0.01) following combined Tat and morphine exposure (Table 1). Unlike the results with combined treatment, morphine treatment alone suppressed c-Rel DNA binding activity to levels below the limit of detection at 4 h, but not at 24 h (0.63±0.01) (Table 1). On the other hand, exposure to Tat by itself transiently augmented c-Rel activity at 4 h (2.49±0.58) with activity returning to control levels by 24 h (0.87±0.04) (Table 1). Of great interest, c-Rel is a member of the NF-κB/Rel family of transcription factors that are known to be involved in regulating expression of MCP-1 and other chemokine genes in a variety of both human and mouse tissues [38], [39] including astrocytes [40].

**Table 1:** Effects of morphine and/or Tat on c-Rel DNA binding activity in striatal astrocytes after 4 or 24 h continuous exposure.

Treatment	DNA binding activity (fold-change from control)			
	Morphine	Tat	Morphine +Tat	
4 h	not detected	2.49±0.58	1.91±0.13*	
24 h	0.63±0.01	0.87±0.04	1.85±0.01*	

* *P*<0.05; significant changes in c-Rel DNA binding activity were noted at 4 h or 24 h following morphine and/or Tat treatment using Kruskal-Wallis nonparametric ANOVA.

Values represent the mean±SEM of the fold-change in transcription factor binding relative to control astrocytes.

### NF-κB Inhibition Reduces Proinflammatory Cytokine and Chemokine Production

**Figure 1:**
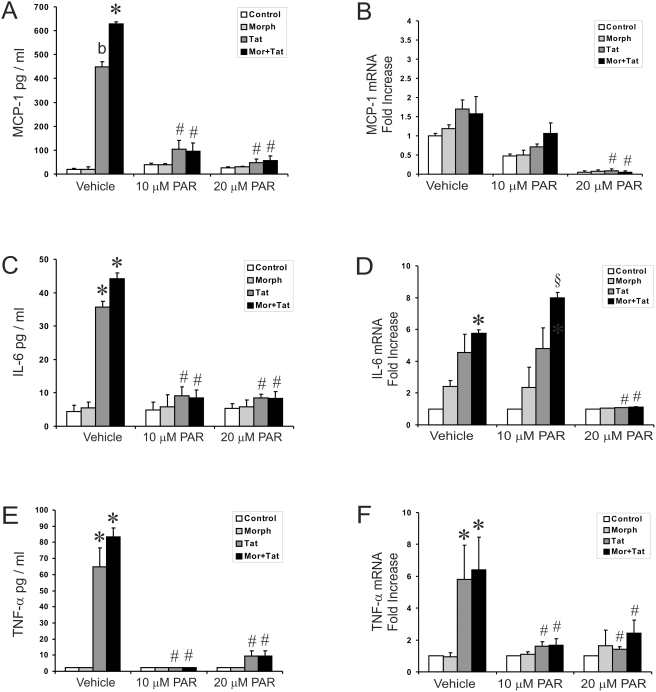
Effects of parthenolide on Tat±morphine-induced increases in MCP-1, IL-6 and TNF-α protein and mRNA levels. Astrocytes were pretreated with 10 or 20 µM of the NF-κB inhibitor, parthenolide (PAR), for 15–20 min followed by incubation with HIV-Tat±morphine (Morph or MOR) for 12 h (A,C,E) or 24 h (B,D,F). Parthenolide exposure inhibited Tat±morphine-induced increases in the release of MCP-1, IL-6, and TNF-α by ELISA at 12 h (A,C,E). Parthenolide exposure (20 µM) caused a significant and sustained reduction in Tat-induced increases in MCP-1, IL-6, and TNF-α mRNA levels at 24 h—that was largely unaffected by coadministering morphine (B,D,F). Values are given as the mean±SEM and representative of at least 3–5 independent experiments (*P<0.05 vs. vehicle-treated controls; ^b^ *P*<0.05 vs. controls or Tat±morphine treatment without PAR; ^#^ *P*<0.05 vs. Tat±morphine treatment without PAR; ^§^ *P*<0.05, vs. Tat+morphine-treated cells exposed to vehicle or 20 µM PAR).

**Figure 2:**
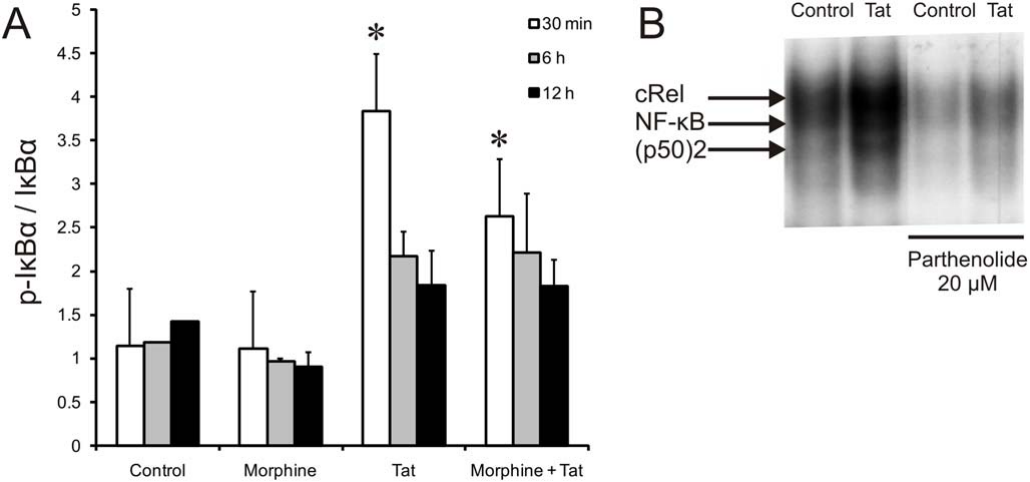
Tat phosphorylates IκBα in astrocytes. To verify that IκBα is phosphorylated in our cell culture system, astrocytes were treated with Tat alone or in combination with morphine for 30 min, 2 and 6 h (A). Increases in IκBα phosphorylation were noted in cell lysates treated with Tat±morphine at 30 min compared to treatment with vehicle or morphine alone (*P<0.05 vs. controls at 30 min). After 6 h, the amount of phosphorylated IκBα was equal in cell lysates treated with Tat alone or Tat plus morphine (A). Down regulation of NF-κB (p65), c-Rel and p50 by parthenolide. Nuclear extracts from cells treated with or without Tat±parthenolide for 24 h were subjected to electromobility shift assay (EMSA) with labeled NF-κB oligonucleotide (B). Nuclear extracts containing parthenolide showed a down regulation in NF-κB DNA binding activity as compared to the DNA binding activity of nuclear extract without parthenolide.

To provide evidence that NF-κB regulates MCP-1, IL-6 and TNF-α production in astrocytes stimulated with HIV-1 Tat±morphine, cultures were pretreated with the NF-κB inhibitor, parthenolide. The anti-inflammatory sesquiterpene lactone, parthenolide, is known to impair the activity of NF-κB by binding directly to and inhibiting the IκB kinase (IκK) complex, thereby preventing the degradation of the inhibitory protein, IκB [41], [42]. Parthenolide was diluted in culture medium and added to cells at different concentrations 15–20 min prior to the addition of Tat and morphine, either alone or in combination. Pretreatment with both 10 µM and 20 µM parthenolide inhibited Tat±morphine-induced increases in TNF-α, MCP-1, and IL-6 release at 12 h (Fig. 1A,C,E). The 12 h time point was chosen to better show that parthenolide causes a sustained diminution in cytokine secretion. Real time-PCR detection of MCP-1, IL-6 and TNF-α mRNA showed that pre-treatment with 20 µM parthenolide completely abolished cytokine mRNA levels—irrespective of the Tat or morphine treatment at 24 h (Fig. 1B,D,F). The 24 h time point was chosen to better show that parthenolide at higher concentrations causes a sustained diminution in mRNA levels. We also noted that the lower concentrations of parthenolide reduced MCP-1, IL-6 and TNF-α release, but only partially inhibited IL-6 and MCP-1 mRNA levels, suggesting the possibility that TNF-α triggers the subsequent release of MCP-1 and IL-6 [43]. NF-κB activation is accompanied by nuclear translocation and upregulation of DNA binding activity. The translocation of NF-κB is usually dependent on the phosphorylation and degradation of IκBα. To verify that IκBα is phosphorylated in our cell culture system, astrocytes were treated with Tat alone or in combination with morphine for 30 min, 2 or 6 h. As shown in Fig. 2A, exposure to Tat caused an initial burst of IκBα phosphorylation at 30 min that gradually declined to levels similar to Tat combined with morphine at 6 and 12 h. Indeed, 6 and 12 h time points were chosen because we found cytokine release is most pronounced during this time in our culture supernatant. Treatment with morphine alone did not affect IκBα levels compared to control cultures, suggesting that Tat alone was responsible for increasing IκBα phosphorylation. Next, to determine whether down regulation of MCP-1, IL-6 and TNF-α by parthenolide is mediated through the sustained inhibition of DNA-binding activities of NF-κB factors, nuclear extracts from cells treated with or without the inhibitor for 24 h were subjected to electrophoretic mobility shift assay (EMSA) (Fig 2B). Parthenolide treatment markedly decreased NF-κB (p50/p65), p50 homodimer, and c-Rel DNA binding activity compared to cells that lacked the inhibitor. Binding specificity was tested using unlabeled wild type- and mutant kB-oligonucleotides as competitors. Wild type κB oligonucleotide, but not mutant oligonucleotide, blocked formation of all three complexes (data not shown). Parthenolide largely blocked NF-κB transcriptional activity, which leads to a reduced cytokine release. The data suggest that transient increases in the activation of NF-κB are required for Tat±morphine-induced increases in cytokines.

### Tat±Morphine Triggers NF-κB/p65 Nucleocytoplasmic Shuttling

**Figure 3:**
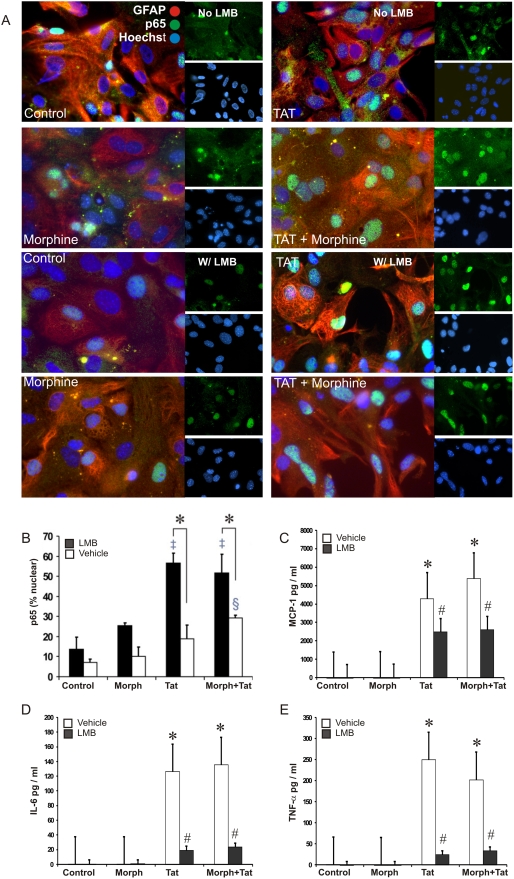
Tat±morphine-induced alterations in cytoplasmic-nuclear shuttling of the p65 subunit of NF-κB regulate cytokine production by astrocytes. Cells were pretreated with vehicle or leptomycin B (LMB) followed by 30 min, 2 or 6 h treatment (only data at 2 h are shown) with Tat±morphine (Morph) and analyzed by immunofluorescence microscopy to determine the proportion of GFAP (red fluorescence) cells with p65(RelA; green fluorescence) subunit present within their nuclei (labeled with Hoechst 33258; blue fluorescence) (A). GFAP^+^ cells containing p65 in the nucleus were counted and divided by the total number of GFAP^+^ cells (B). Tat alone or in combination with morphine increased the trafficking of the p65(RelA) subunit of NF-κB into the nucleus (A–B), while blocking nuclear export with LMB increases the accumulation of p65(RelA) subunits in the nucleus (A–B; *P<0.05 vs. vehicle-or LMB-treated controls; ^§^ *P*<0.05 vs. vehicle-treated controls; ^‡^ *P*<0.05 vs. Tat±morphine treatment without LMB). (C–E): LMB attenuates cytokine release caused by Tat±morphine. Analysis of astrocyte conditioned medium by ELISA showed a significant decrease in MCP-1, IL-6 and TNF-α production in astrocytes pretreated with LMB (*P<0.05 vs. vehicle or LMB-treated controls lacking Tat±morphine; ^#^ *P*<0.05 vs. Tat+morphine treated astrocytes without LMB). Data represent the mean±SEM of 3 separate experiments at 6 h.

The NF-κB complex must shuttle into the nucleus before it can influence transcription. NF-κB shuttling has been shown to be involved in triggering chemokine production in astrocytes [31]. To determine the extent to which Tat±morphine affected the rate of NF-κB nuclear translocation and cytokine gene expression in our system, astrocytes were pretreated with a nuclear export inhibitor, leptomycin B (LMB), and the effects of HIV-Tat±morphine were assessed at 30 min, 2 and 6 h. Treatment with Tat alone or in combination with morphine caused time-dependent increases in the percentage of cells expressing p65 (RelA) within their nuclei as compared to vehicle (control) or morphine-treated cells (Fig. 3 A). A ratio was determined by counting every GFAP^+^ cell containing the p65 in the nucleus divided by the total number of cells (Fig. 3B). As expected, in astrocytes pretreated with 20 nM LMB, we observed that with longer treatment times an increasing percentage of cells retained nuclear p65 (RelA), until only trace amounts of p65 were detected in the cytoplasm (Fig. 3 A–B). Cytokine secretion was also affected by LMB. Supernatants from astrocytes treated with Tat±morphine showed a significant decrease in MCP-1 IL-6 and TNF-α (Fig. 3C–E), in the presence of LMB. Moreover, cytokine release was inhibited despite the retention of the p65 (RelA) subunit in the nucleus. These data imply that NF-κB enters the nucleus and functions in cytokine production, and that continued recycling of NF-κB is necessary for sustained cytokine transcription and expression. Thus, we propose that combined Tat and morphine cause a sustained cytokine expression/release in astrocytes through the sustained phosphorylation of p65 (RelA) and enhanced nucleocytoplasmic shuttling of NF-κB.

### [Ca^2+^]_i_ Chelation Abrogates Tat±Morphine Induced Cytokine Release by Astrocytes

**Figure 4:**
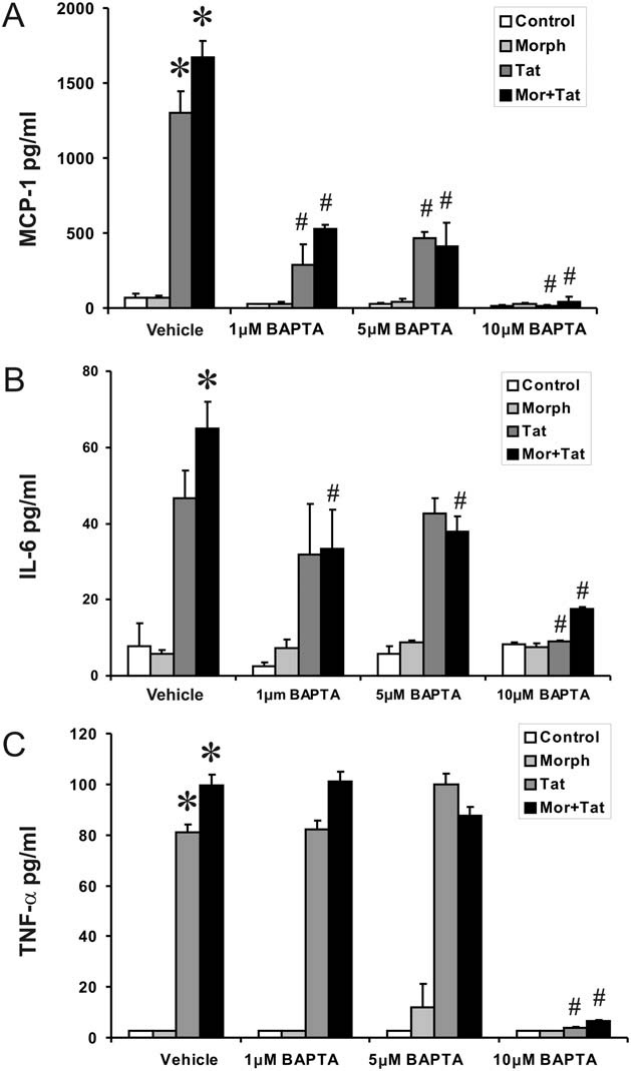
Effects of BAPTA on Tat±morphine-induced cytokine release by astrocytes. Buffering [Ca^2+^]_i_ blocked Tat±morphine (Morph or MOR) induced cytokine release by astrocytes at 6 h. Stimulation with Tat±morphine caused significant increases in MCP-1, IL-6, and TNF-α production compared with untreated controls (A–C). The Ca^2+^ chelator BAPTA caused significant, concentration-dependent reductions in the release of MCP-1, IL-6 and TNF-α by astrocytes. Results are representative of 3 independent experiments, and the data are given as the mean±SEM at 6 h (*P<0.05 vs. vehicle-treated controls; ^#^ *P*<0.05 vs. identical treatment without BAPTA).

**Figure 5:**
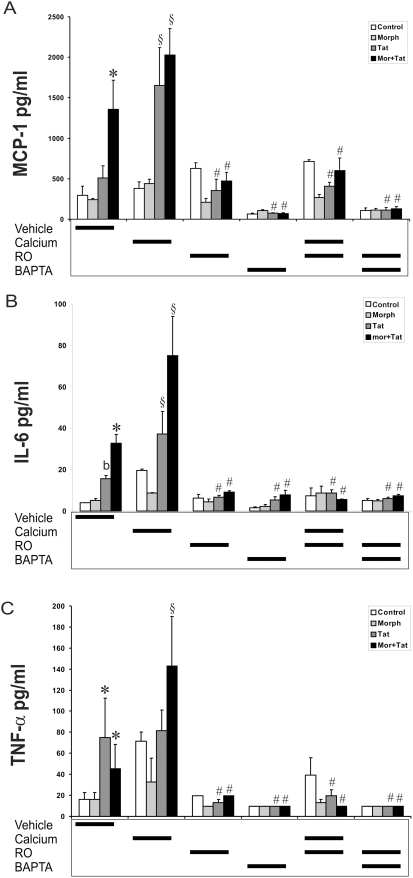
Blocking NF-κB nuclear import prevents Tat±morphine-induced cytokine release by astrocytes (A–C). Astrocytes were pretreated with Ro106-9920 (RO; 10 µM), which prevents the import of NF-κB into the nucleus, for 15 min followed by the addition of Tat±morphine for up to 6 h. RO exposure significantly decreased Tat±morphine-evoked cytokine release by astrocytes. In addition, RO attenuated Tat-induced increases in the presence of high levels of Ca^2+^, which is induced by treating astrocytes with 5 mM Ca^2+^ outside the cell ([Ca^2+^]_o_). Despite increases in Ca^2+^, preventing the import of NF-κB into the nucleus completely prevented Tat±morphine-induced increases in cytokine release (*P<0.05 vs. vehicle-treated controls; ^b^ *P*<0.05 vs. control, morphine, or Tat+morphine-treated controls; ^§^ *P*<0.05, vehicle vs. 5 mM [Ca^2+^]_o_-treatment; ^#^ *P*<0.05 vs. Tat±morphine treatment without RO, BAPTA, or 5 mM [Ca^2+^]_o_).

To determine whether upsurges in [Ca^2+^]_i_ affected Tat±morphine-induced cytokine release by astrocytes, [Ca^2+^]_i_ was buffered by pretreating astrocytes with the intracellular calcium chelator, BAPTA/AM. Astrocytes were pretreated with varied concentrations of Ca^2+^ or the Ca^2+^ chelator BAPTA/AM in the medium for 20 min followed by incubation with Tat±morphine for up to 6 h. Stimulation with Tat alone or in combination with morphine led to increases in MCP-1, IL-6 and TNF-α production compared with untreated controls (Fig 4A–C). Pretreatment with 1 or 5 µM BAPTA/AM partially inhibited MCP-1 and IL-6, while BAPTA/AM at 10 µM completely abolished production of morphine and Tat-induced increases in MCP-1, IL-6 and TNF-α. This suggests that TNF-α was less affected by decreases in intracellular calcium than MCP-1 and IL-6, and further implicates TNF-α as a trigger in the release of other cytokines. Furthermore, the release of MCP-1 and IL-6 is highly calcium-dependent. Next, Ca^2+^ was added exogenously to each well, and increasing Ca^2+^ concentration outside the cell ([Ca^2+^]_o_), markedly augmented Tat±morphine-evoked MCP-1 and IL-6, but not TNF-α release (Fig. 5A–C). Again in this experiment, TNF-α was less affected by changes in [Ca^2+^]_i_ compared to MCP-1 and IL-6. Interestingly, however, increasing [Ca^2+^]_i_ by itself is not sufficient to increase cytokine release. Despite increases in [Ca^2+^]_i_, morphine treatment alone did not increase cytokine release suggesting that an additional signal independent from Ca^2+^, such as the phosphorylation of NF-κB or I-kappa-B-kinase (IKK), must also occur in concert with increases in [Ca^2+^]_i_. Thus, while increasing [Ca^2+^]_i_ may be necessary, it is not sufficient to induce cytokine release by astrocytes. Collectively, our findings suggest that Tat alone is sufficient to trigger the [Ca^2+^]_i_-dependent signal and NF-κB activity, while morphine is not. However, in combination with Tat, morphine can modulate Tat-induced cytokine release through a Ca^2+^-dependent mechanism involving the NF-κB pathway.

### Inhibition of IκBα Degradation and NF-κB-Dependent Cytokine Expression in Astrocytes

Next, we investigated whether the association with IκBα regulates the activity of NF-κB and cytokine production in our system. We used Ro106-9920 to target the ubiquitination of IκBα and inhibit the dissociation of NF-κB/IκBα complex [44], and prevent the nuclear import of NF-κB. Astrocytes were pretreated with Ro106-9920 for 15–20 min prior to the addition of Tat, morphine, or Tat plus morphine for 6 h. Ro106-9920 (10 µM) treatment significantly decreased Tat±morphine-evoked cytokine release by astrocytes, highlighting the important role of NF-κB in cytokine secretion (Fig. 5A–C). Next, it was determined whether inhibiting the dissociation of the NF-κB/IκBα complex could still attenuate Tat-induced cytokine increases, even in the presence of high levels of Ca^2+^. Despite increases in [Ca^2+^]_i_ (induced by treating astrocytes with 5 mM Ca^2+^), inhibiting the activation of NF-κB completely prevented Tat±morphine-induced increases in cytokine release (Fig 5 A–C; Cal+RO). No changes in cytokine production were seen in astrocytes treated with BAPTA/AM or with BAPTA/AM+Ro106-9920 (Fig 5 A–C), suggesting that the IkBα-NF-kB complex is located downstream of Ca^2+^ signaling events. Cytotoxicity was not observed in astrocytes with any of the above treatments after 2–6 h incubation (not shown). Altogether, these results suggest that the degradation of IκB proteins is necessary for NF-κB nuclear translocation and cytokine gene upregulation in astrocytes treated with Tat±morphine. Moreover, the fact that Ro106-9920 blocked cytokine release even in the presence of high [Ca^2+^]_i_ suggests a strong involvement of NF-κB, and imply that once this pathway is shut down it is virtually impossible to generate cytokines irrespective of Ca^2+^.

### Ca^2+^ Transiently Induces NF-κB Activation and Nuclear Translocation

**Figure 6:**
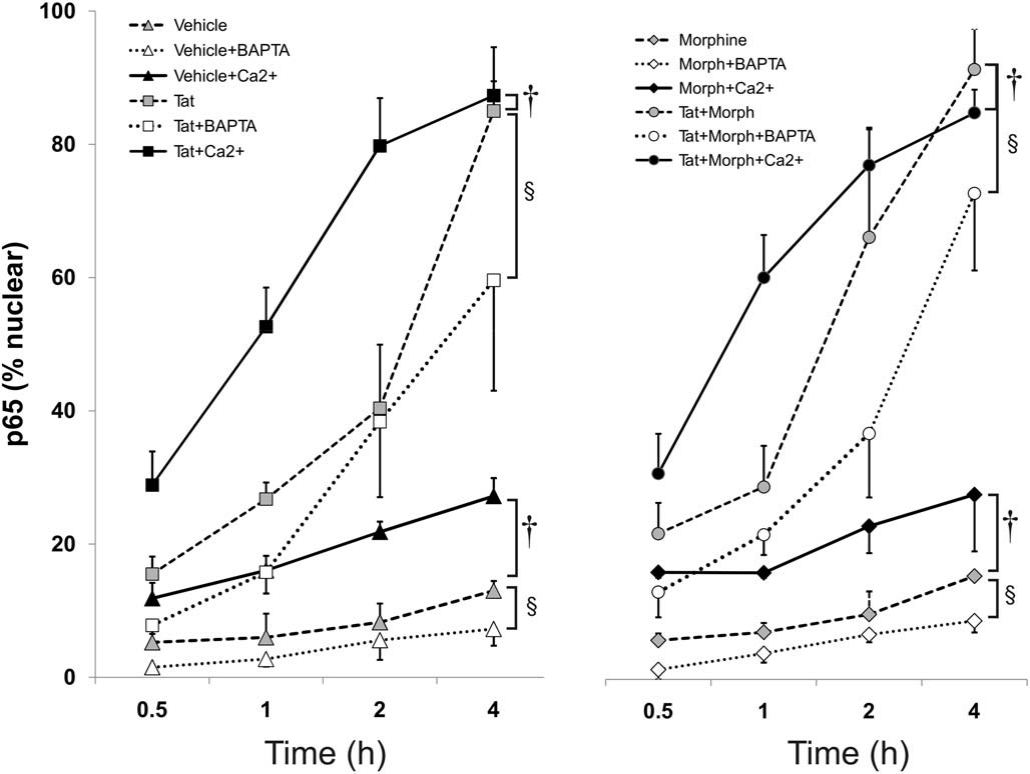
Ca^2+^ modulates morphine and/or HIV-1 Tat-induced trafficking of the p65 (RelA) subunit of NF-κB into the nucleus of astrocytes. Chelating [Ca^2+^]_i_ with 10 µM BAPTA significantly delays Tat and/or morphine-(Morph or MOR) induced nuclear import of p65(RelA), while artificially elevating [Ca^2+^]_i_ by excessive (5 mM) Ca^2+^ outside the cell ([Ca^2+^]_o_), accelerates p65 subunit trafficking into the nucleus. Note that p65 nuclear shuttling occurs both in vehicle and in BAPTA-treated astrocytes, suggesting that nucleocytoplasmic shuttling occurs continuously. It also infers that other events such as the phosphorylation of specific NF-κB subunits are operative (^§^ *P*<0.05, vehicle vs. BAPTA -treated cells; ^†^ *P*<0.05, control vs. 5 mM [Ca^2+^]_o_-treated cells).

Lastly, we investigated whether Ca^2+^ chelation interferes with Tat±morphine-induced NF-κB activation and nuclear translocation. Astrocyte cultures were pretreated with vehicle (culture medium), BAPTA/AM (10 µM), or excess Ca^2+^ outside (5 mM), for 15–20 min before exposure to vehicle, morphine (Morph) or Tat±morphine for 30 min, or 1, 2, or 4 h. The proportion of astrocytes stained with p65 (RelA)-immunoreactive nuclei was then determined. Tat±morphine markedly increased the translocation of the p65 (RelA) subunit of NF-κB into the nucleus of astrocytes, while chelating Ca^2+^ with BAPTA significantly attenuates p65 (RelA) nuclear entry in vehicle (control), morphine, or Tat±morphine exposed astrocytes (Fig. 6). Conversely, adding excess extracellular Ca^2+^ accelerated translocation of p65 (RelA) into the nucleus in all treated groups. The effects were time-dependent and the combined effects of Tat and morphine were more robust than those seen with Tat alone. Detection of p65 (RelA) in the nuclei of about 5% of astrocytes at 30 min and the ability of BAPTA/AM to markedly reduce p65 (RelA) nuclear labeling in vehicle-treated control cultures suggested that p65 (RelA)/NF-κB was being tonically activated at low levels in our cell cultures. Buffering [Ca^2+^]_i_ with BAPTA/AM significantly delays nuclear import, while artificially elevating Ca^2+^ by excessive (5 mM) Ca^2+^ outside the cell accelerates p65 (RelA) subunit trafficking into the nucleus. Note that some p65 (RelA) nuclear shuttling occurs in vehicle-treated astrocytes suggesting that tonic levels of nucleocytoplasmic shuttling occur and that other events such as the phosphorylation of specific NF-κB subunits are operative (Fig. 6). Thus, calcium affects p65 (RelA)/NF-κB activity by modulating its trafficking into the nucleus.

## Discussion

Our previous data showed that opiates can exacerbate the release of cytokines in HIV-1 Tat protein exposed astrocytes [17]. The interactive effects are time-dependent, differ among affected cytokines, and mediated through μ-opioid receptor-expressing subpopulations of astrocytes. We also showed that μ opioid receptor activation or exposure to Tat increased Ca^2+^, while causing synergistic increases in [Ca^2+^]_i_ in combination [17]. Prior studies indicated that morphine elevated [Ca^2+^]_i_ in astrocytes through several different pathways including influx through voltage-dependent L-type Ca^2+^ channels and mobilization through IP_3_-dependent pools, with subsequent Ca^2+^-induced Ca^2+^ release (CICR or regenerative Ca^2+^) from internal stores [37]. Based on long-standing evidence of the involvement of NF-κB in the production of inflammatory mediators, we predicted that opiates would potentiate the effects of HIV-1 Tat on proinflammatory cytokine production by astrocytes through signals converging at NF-κB. To test this hypothesis, we examined whether exposure to HIV-1 Tat±morphine modulates the activation of NF-κB and whether this in turn was responsible for IL-6, TNF-α, and MCP-1 by astrocytes. In addition, we also explored the extent to which Tat and opiate-induced changes in [Ca^2+^]_i_ modulate activation of the NF-κB pathway. In the present study, Tat±morphine increased IkBα phosphorylation, the rate of p65 translocation into the nucleus, and NF-κB binding by EMSA, while the NF-κB inhibitor parthenolide eliminated cytokine release in Tat±morphine treated astrocytes. In addition, manipulating [Ca^2+^]_i_ modified both Tat±morphine-evoked p65/NF-kB trafficking into the nucleus and cytokine release to a significant extent. Chelating [Ca^2+^]_i_ prevented Tat±morphine-induced p65/NF-κB nuclear translocation and cytokine release, while increasing [Ca^2+^]_i_ above baseline levels exacerbated these effects. Taken together, the results suggest that Tat induces cytokine production by astrocytes through a pathway dependent on [Ca^2+^]_i_, NF-κB nuclear translocation and transcriptional activity, while morphine appears to enhance the effects of Tat through a convergent signal involving [Ca^2+^]_i_ and the subsequent rate of p65/NF-κB shuttling in to the nucleus.

**Figure 7:**
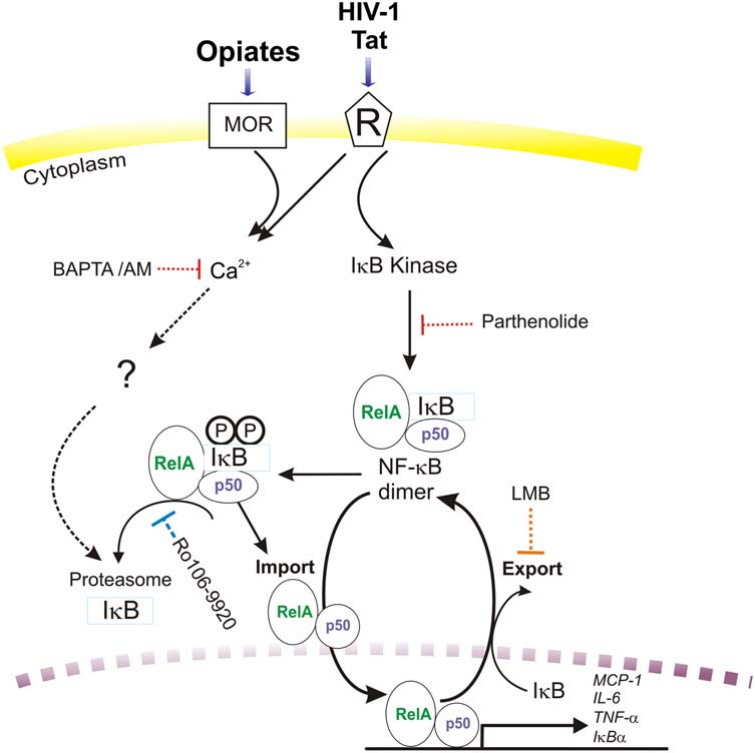
Proposed model for the involvement of NF-κB signaling in the regulation of cytokine and chemokine in Tat±morphine treated astrocytes. Arrows indicate activation of the signaling pathway; the “┤” signs indicate pathway inhibition. Exposure to Tat alone increases IκB phosphorylation/degradation and NF-κB transcriptional activity, and additionally increases [Ca^2+^]_i_ in astrocytes [48]. Although morphine treatment alone fails to significantly activate NF-κB in astrocytes, in combination with Tat it can potentiate Tat-induced increases in [Ca^2+^]_i_ at sites upstream of NF-κB [37]. How [Ca^2+^]_i_ affects IκB degradation in the present study is uncertain (?), but may involve increased oxidative stress [55], and decreased proteasome activity.

Based on data from this study, we propose that Tat increases cytokine production because it can both elevate [Ca^2+^]_i_ and activate NF-κB. The addition of exogenous calcium increases intracellular calcium, after functionally depleting energy-dependent calcium pumps, and overfilling of endoplasmic reticulum (ER) (and other, including nuclear stores), an overfilling of ER stores would likely boost the ability of Tat to induce cytokine release. Therefore, Tat is sufficient for cytokine release by astrocytes. Opiates alone are not sufficient to drive increases in cytokine release because they do not appear to activate NF-κB in astrocytes. In fact, morphine fails to act even when [Ca^2+^]_i_ is artificially elevated by high levels of extracellular Ca^2+^. Opiates, however, can potentiate the effects of Tat through further increases in [Ca^2+^]_i_ at sites upstream of NF-κB (summarized in Fig. 7). We speculate that increases in [Ca^2+^]_i_ are accompanied by a Ca^2+^-independent signal that likely involves the phosphorylation and targeted ubiquitination of IκBα, which frees NF-κB allowing it to translocate into the nucleus.

Studies using the Ca^2+^ chelator BAPTA/AM provide circumstantial evidence that [Ca^2+^]_i_ modulate NF-κB activity. The possible regulation of NF-κB pathway by Ca^2+^ signaling was indirectly implied in studies showing that release of MCP-1, IL-6 and to a lesser extend TNF-α was adversely affected by the Ca^2+^ chelator BAPTA. To explore whether Ca^2+^ affects the activation and translocation of NF-κB, we incubated astrocytes with BAPTA/AM followed by treatment with Tat±morphine. BAPTA significantly reduced MCP-1 and IL-6 expression in a concentration-dependent manner while production of TNF-α was reduced but only at the highest concentration of BAPTA, inferring differential thresholds of Ca^2+^ sensitivity for TNF-α versus MCP-1 and IL-6. Alternatively, it may be possible that TNF-α serves as an initiator of cytokine release. In fact, others have shown that RANTES stimulates the release of TNF-α which subsequently participates in the amplification of the astrocyte inflammatory cascade by stimulating MCP-1 production [16], [45]. Interestingly, we previously found that morphine could exacerbate Tat-induced increases in the release of RANTES and MCP-1, but not TNF-α, by striatal mouse astrocytes [17]. The discrepancies in the data might be explained by differences in the techniques used to detect cytokines (Pananomics/Raybio antibody arrays vs. ELISA) or differences in the phenotype and composition of astrocyte-enriched cultures obtained from postnatal day 0–1 vs. postnatal day 4 mice. For example, the proportion of microglia and oligodendroglia increase, while the percentage of glial precursors decreases with maturation. Collectively, the results suggest that TNF-α production is regulated differently from MCP-1 and IL-6 and differs in its response to morphine. Moreover, we found that buffering [Ca^2+^]_i_ reduced the trafficking of the p65 (RelA) subunit of NF-κB and reduced Tat±morphine-induced increases in p65 nuclear translocation. The results of our data imply that [Ca^2+^]_i_ can regulate the activation and nuclear translocation of NF-κB. Our results agree with findings by others that the induction of NF-κB activity can be regulated by Ca^2+^, and that Ca^2+^ oscillations increase the efficiency and specificity of the expression of various genes [46]. Lilienbaum and Israël [47] showed that transcriptional activity of NF-κB in primary neurons can be regulated by a complex network, involving Ras, PI3K, and Akt, all of which are controlled by Ca^2+^. Their results show that instead of a single signaling pathway, NF-κB activity in neurons is regulated by integrated cross talk of multiple transduction cascades.

The specific pathway(s) by which Ca^2+^ is activated in astrocyte cultures following treatment with Tat alone or in combination with morphine is unclear. One possibility is that BAPTA suppresses the activation of NF-κB by buffering cytoplasmic Ca^2+^ increases that are necessary for the phosphorylation of IκK. Although we cannot say whether the Ca^2+^ increases originate from internal stores and/or influx of extracellular Ca^2+^ [37], it is possible that the signal involves the convergence of multiple independent Ca^2+^ pathways. For example, dantrolene can modify Tat and morphine-evoked cytokine release (El-Hage, unpublished) suggesting that CICR propagates that signal. Alternatively, Ca^2+^ mobilization via IP_3_-dependent internal stores or Ca^2+^ influx via L-type Ca^2+^ channels could be potentially involved since both are independently affected by morphine [37] or Tat [48], [49].

NF-κB activity can be regulated at several levels, including through alterations in cytoplasmic-nuclear shuttling. To assess the role of trafficking, we pretreated astrocytes with a nuclear export inhibitor, leptomycin B (LMB). LMB at a low concentration is a potent and specific inhibitor of many proteins including the HIV-1 Rev protein, MAPK/ERK, and the nuclear export protein CRM1 [50], [51]. In the present study, treatment with LMB blocked cytokine production, despite the continued presence of nuclear p65 (RelA), indicating that NF-κB turnover is essential for cytokine release by astrocytes exposed to Tat±morphine. Our data agrees with others who have shown that NF-κB shuttling is a mechanism that is involved in the induction of chemokines in astrocytes [31], and appears to constitutively occur at low levels even in non-activated cells [52]. The small molecule, Ro106-9920, was used to further investigate the signal transduction mechanism involved in NF-κB-induced cytokine production in astrocytes treated with Tat±morphine. Perhaps not surprisingly, Ro106-9920 prevented cytokine release from Tat or Tat plus morphine-treated astrocytes, since this molecule is known to inhibit IκBα ubiquitination and thereby prevent NF-κB dimers from forming and translocating into the nucleus. Our results indicate that IκBα ubiquitination is essential for nuclear translocation of the NF-κB dimeric complex leading to the regulation of cytokine production.

TNF-α and IL-1 trigger a classical pathway involving the activation of the IKK complex leading to the phosphorylation and then the ubiquitination and degradation of IκBα, allowing NF-κB to translocate to the nucleus. This pathway is crucial for the activation of innate immunity and inflammation [29], [53], although alternative pathways have been proposed [33]. NF-κB activation can also be modulated by G-protein coupled receptors such as the μ opioid receptor through a variety of upstream events including the cAMP/PKA/CREB, PI3K/Akt/IKK and PLC/PKC/IκK pathways [54]. Moreover, increased reactive oxygen species (ROS) can activate NF-κB. Once primed by oxidative stress, increases in [Ca^2+^]_i_ can exacerbate NF-κB transcriptional activity [55], which is similar to our preliminary findings in astrocytes and infers that Ca^2+^-dependent increases in ROS could mediate the effects of Tat±morphine. Preliminary data indicates that morphine significantly increases oxyradical production in Tat-exposed astrocytes. It is important to emphasize that NF-κB can serve dramatically distinct functions in different neural cell types. For example, while NF-κB activation in immune cells such as macrophages/microglia herald inflammation, oxyradical production, and cell stress, NF-κB-dependent transcription in neurons has entirely different consequences promoting synaptic plasticity, [Ca^2+^]_i_ homeostasis, and prolonged viability [56]–[58]. For this reason, it may be an overgeneralization to assume that NF-κB functions are the same in astrocytes and macrophages/microglia despite superficial similarities in cytokine release. Furthermore, the consequences of μ opioid receptor activation are so dissimilar in astrocytes from other cell types [59], that it would be challenging to predict the nature of converging opiate-Tat toxic signals without empirical testing in astrocytes. Therefore, additional studies are needed to clarify a potential relationship between elevated Ca^2+^, ROS, the potential inhibition of proteasome proteolysis, and NF-κB transcriptional activity in astrocytes.

Irrespective of the exact mechanisms involved, here we show that exposure to Tat alone increases IκB phosphorylation/degradation and NF-κB transcriptional activity while also increasing [Ca^2+^]_i_ in astrocytes [48]. On the other hand, morphine treatment alone fails to significantly activate NF-κB in astrocytes, and causes modest increases in [Ca^2+^]_i_. However, morphine in the presence of Tat potentiates Tat-induced cytokine release by increasing [Ca^2+^]_i_ at sites upstream of NF-κB, which increases NF-κB activation and cytokine production. The unique proinflammatory response of astroglia to combined Tat and morphine exposure, which is mediated by NF-κB and modified by changes in Ca^2+^ homeostasis, might explain why the CNS is especially vulnerable to the combined effects of opiate abuse and human immunodeficiency virus encephalitis (HIVE) [60]. Although additional studies are needed to determine how Ca^2+^ modulates NF-κB activation, our results suggest that astrocytes are significant in orchestrating the deleterious effects of opiate abuse in the CNS of HIV-1 infected individuals.
